# Safety Aspects, Tolerability and Modeling of Retinofugal Alternating Current Stimulation

**DOI:** 10.3389/fnins.2019.00783

**Published:** 2019-08-07

**Authors:** Linus Haberbosch, Abhishek Datta, Chris Thomas, Andreas Jooß, Arvid Köhn, Maria Rönnefarth, Michael Scholz, Stephan A. Brandt, Sein Schmidt

**Affiliations:** ^1^Department of Neurology, Charité – Universitätsmedizin Berlin, Berlin, Germany; ^2^Department of Endocrinology, Diabetes and Metabolism, Charité – Universitätsmedizin Berlin, Berlin, Germany; ^3^Research and Development, Soterix Medical, New York, NY, United States; ^4^Berlin Institute of Health (BIH), Berlin, Germany; ^5^Neural Information Processing Group, Technical University of Berlin, Berlin, Germany

**Keywords:** retinofugal alternating current stimulation, electrical stimulation, feasibility, tolerability, safety, adverse events, finite element modeling

## Abstract

**Background:**

While alternating current stimulation (ACS) is gaining relevance as a tool in research and approaching clinical applications, its mechanisms of action remain unclear. A review by Schutter and colleagues argues for a retinal origin of transcranial ACS’ neuromodulatory effects. Interestingly, there is an alternative application form of ACS specifically targeting α-oscillations in the visual cortex via periorbital electrodes (retinofugal alternating current stimulation, rACS). To further compare these two methods and investigate retinal effects of ACS, we first aim to establish the safety and tolerability of rACS.

**Objective:**

The goal of our research was to evaluate the safety of rACS via finite-element modeling, theoretical safety limits and subjective report.

**Methods:**

20 healthy subjects were stimulated with rACS as well as photic stimulation and reported adverse events following stimulation. We analyzed stimulation parameters at electrode level as well as distributed metric estimates from an ultra-high spatial resolution magnetic resonance imaging (MRI)-derived finite element human head model and compared them to existing safety limits.

**Results:**

Topographical modeling revealed the highest current densities in the anterior visual pathway, particularly retina and optic nerve. Stimulation parameters and finite element modeling estimates of rACS were found to be well below existing safety limits. No serious adverse events occurred.

**Conclusion:**

Our findings are in line with existing safety guidelines for retinal and neural damage and establish the tolerability and feasibility of rACS. In comparison to tACS, retinofugal stimulation of the visual cortex provides an anatomically circumscribed model to systematically study the mechanisms of action of ACS.

## Introduction

Non-invasive brain stimulation (NiBS) is an effective method for research, as well as a promising tool for therapy in cognitive and clinical neuroscience (Paulus, 2003; Hallett, 2007; Liew et al., 2014). Its effects range from direct brief modification of neural activity to long lasting recovery maximization following neural injury (Hallett, 2005; Talelli and Rothwell, 2006; Hummel et al., 2008; Sandrini and Cohen, 2013). Recently, transcranial alternating current stimulation (tACS), characterized by oscillatory low-voltage stimulation, showed promising effects on the motor system (Feurra et al., 2011, 2013), motor performance (Pogosyan et al., 2009; Joundi et al., 2012), memory (Marshall et al., 2006; Polania et al., 2012), higher order cognition (Santarnecchi et al., 2013, 2016) and tremor (Brittain et al., 2013). Despite these encouraging results, tACS’ mechanisms of action remain unclear (Zaghi et al., 2010) and a retinal contribution to its effects on neural synchrony is still being discussed (Schutter, 2016).

Retinofugal alternating current stimulation (rACS) is a comparably novel form of alternating current stimulation (ACS). In contrast to tACS, rACS is characterized by transmission along retinofugal tracts terminating predominantly in cortical visual areas and neuromodulation of central rhythms (Gall et al., 2011; Schmidt et al., 2013a). While differing from other forms of NiBS in regard to stimulation site, rACS shares its use of alternating current and effects on the intrinsic frequencies of the visual system with tACS (Schmidt et al., 2013a; Haberbosch et al., 2019). Moreover, in comparison to other forms of NiBS (namely, most types of tES) with diffusely induced electric fields (EF) throughout large parts of the brain (Peterchev et al., 2012), rACS affects the well-defined retinofugal pathway (Rager and Singer, 1998) for stimulation confined to the visual system. Thus, rACS renders a unique means to study mechanisms underlying NiBS as it physiologically affects the circumscribed primary visual cortex with separate input from each eye.

Before any novel method can be employed to its full potential or compared with other methodologies, establishing its safety and tolerability is critically important (Bath et al., 2014). The lack of knowledge of safety parameters could culminate in ineffective or even hazardous use (Antal et al., 2008; Bath et al., 2014). While ineffective stimulation could lead to incoherent findings regarding stimulation effects, effective but hazardous use could possibly result in severe adverse events and lasting damages in stimulation subjects. As rACS is used for research purposes, its safety as well as tolerability has to be determined rigorously.

Refraining from potentially dangerous invasive measures, the safety of a novel NiBS montage should be assessed in several different ways.

Firstly, stimulation parameters can be compared to theoretical safety limits as established for NiBS and neural tissue damage in animal studies (Agnew and McCreery, 1987; Liebetanz et al., 2009; Jackson et al., 2017), which have since been used to assess NiBS safety in human studies (Poreisz et al., 2007; Bikson et al., 2009, 2016). The primarily employed metrics include current density (A/m^2^) and charge density (C/m^2^), although other parameters such as charge per phase (C/ph) have been proposed to account for the shifting polarity of AC stimulation (Nitsche et al., 2003; Merrill et al., 2005).

Secondly, these safety metrics can be modeled onto CNS structures (Datta et al., 2011; Bikson et al., 2016), to determine the possibility of damage at critical locations (Bikson et al., 2016) while accounting for anatomy and electrode position (Bikson et al., 2009; Bikson and Datta, 2012; Peterchev et al., 2012; Saturnino et al., 2015).

Finally, experimental validation of theoretical results by subjective reports of adverse events with validated questionnaires can be acquired (Brunoni et al., 2011). These reports are also instrumental in assessing the tolerability of the novel method.

In this study we hypothesized that rACS is to be considered safe if: (1) Stimulation parameters (current and charge densities at the electrode) are within theoretical safety limits, (2) finite element modeling data shows the same for EF estimates and current densities at eye, retina and cortex, and (3) adverse events do not exceed that of other established stimulation methods in rate as well as severity.

To address the primary hypothesis, the stimulation parameters of rACS were recorded during stimulation and employed for the calculation of safety limits. Ultra-high resolution topographical finite element modeling was performed to identify regions of critical interest and to calculate theoretical safety parameters. Adverse events were identified with an extended adverse events questionnaire developed for tDCS (Brunoni et al., 2011). For direct experimental comparison, we employed simple and safe photic stimulation (PS) (Walker et al., 1944) as the gold-standard method for stimulation of the retinofugal pathway regarding safety and clinical experience (Cobb, 1947; Trenite et al., 1999).

## Materials and Methods

To address the safety profile of rACS, we observed and questioned 20 test subjects during rACS and PS sessions. We assessed cutaneous, retinal and central adverse events and drew a comparison between PS and rACS.

### Participants

We stimulated 20 healthy volunteers (10 men), mean age 25.9 ± 4.95, as part of a study investigating a common framework of action for NiBS. The subjects were interviewed prior to experimentation regarding their state of health. We applied established exclusion criteria for NiBS (Brunoni et al., 2012) and added evidence for photophobia and photosensitive epilepsy. Written informed consent was obtained from all individual participants included in the study. The subjects received financial compensation for their participation. All procedures were performed in accordance with the ethical standards of the Ethics Committee of the Charité – Universitätsmedizin Berlin (“Ethikkommission der Charité – Universitätsmedizin Berlin”) and with the 1964 Declaration of Helsinki and its later amendments. This study adheres to the principles of good scientific practice of the Charité – Universitätsmedizin Berlin (“Grundsätze der Charité zur Sicherung guter wissenschaftlicher Praxis”).

### Retinofugal Alternating Current Stimulation (rACS)

Retinofugal alternating current stimulation was applied via a multi-channel low-voltage stimulation device certified for clinical use, which delivered weak oscillatory current sinus-pulses over four individual periorbital electrodes, respectively (NextWave, Eyetronic, Germany). The four superficial active stimulating electrodes (Grass SAFELEAD^TM^ gold electrodes, Astro-Med, Inc., RI, United States) were contained in foam-padded stimulation goggles and bilaterally made skin contact via small felt buffers (0.35 cm^2^) superior and inferior to the eye. The return electrode (rectangular electrode, 30 × 30 mm polished stainless steel) was fastened on the back of the neck at the midline.

Alternating current was applied at 10 Hz, as ACS has shown robust effects at this frequency (Kanai et al., 2008; Helfrich et al., 2014; Vossen et al., 2015) and gold standard PS typically also employs 10 Hz stimulation (Photic driving) (Walker et al., 1944). Stimulation amplitude was set to 120% phosphene threshold (resulting in 351.69 μA (SD 63.95) peak-to-peak amplitude). The phosphene threshold was determined employing an ascending method of limits (Herrick, 1967) provided by the NextWave software. rACS was delivered in 30 s blocks followed by 30 s pauses over 10 min. The subjects were told to keep their eyes open and focus a fixed point on a white surface in 1 m distance for the duration of the experiment.

To assess the safety parameters of stimulation we additionally calculated the effective amplitude. The effective amplitude of the applied current is defined as the time normed integral of the signal, which simplifies to its mean value for discrete signals as is the case here, since the used stimulator receives an equidistant sampled discrete function as input. In the simplest case of a pure sine wave this simplifies to the following formula:

$$a(\mathrm{eff})=\frac{a_{\text{max}}}{\sqrt{2}}$$

In the case of more complex stimuli such as noise + sine wave or signals with an additional amplitude modulation, the use of peak-to-peak “a(max)” values to describe the resulting electrical power of an electric current stimulation would be misleading.

Regarding charge, we decided to refrain from more complex line integral calculations, and instead used the following simple formula:

Q=I*t

This was done to ensure straightforward comparability of resulting values. It also adds to the rigidity of our safety considerations by rather over-than underestimating the injected charge.

### Photic Stimulation

Photic Stimulation was applied via two 3 × 5 cm multi-color white LEDs contained in the stimulation goggles, which work via red, green and blue LEDs mixing their emissions to form white light. To be able to compare stimulation intensities with rACS, sinusoidal pulses of white light were applied at an intensity of 120% light threshold and with a frequency of 10 Hz. This threshold was also determined by an ascending method of limits and resulted in an average luminous intensity of 1.24 cd (±0.44) for stimulation.

The stimulation was also delivered in 30 s blocks followed by 30 s pauses over 10 min, and the subjects received the same instructions as for rACS.

### Modeling

The ultra-high resolution head and neck model (MIDA: Multimodal Imaging-Based Detailed Anatomical Model) available through the IT’IS Foundation was used in this study (Iacono et al., 2015). The nifti (.nii) color masks from the MIDA model were first processed in MATLAB to re-create segmentation masks based on intensity values. These masks were then imported into Simpleware (Synopsys Ltd., CA, United States) and any errors in continuity and anatomical details were manually corrected for Datta et al. (2012). Masks with similar electrical conductivities were then merged to a single compartment barring the regions of interest (eye structures) in order to perform individual current flow analysis through them. For instance, mandible, teeth, vertebra, skull dipole, skull inner table, skull outer table, hyoid bone were combined with the skull mask but eye retina, choroid, and sclera were treated as individual masks.

**FIGURE 1:**
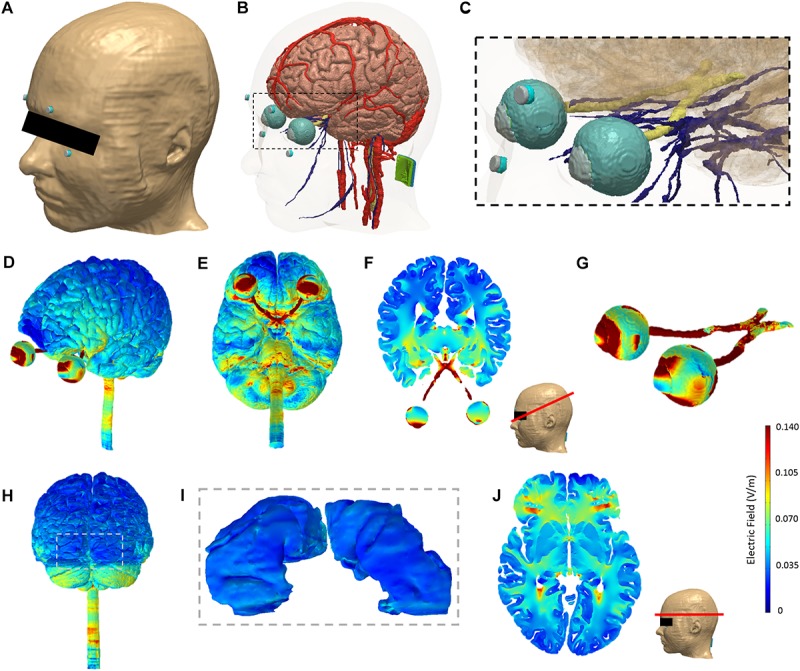
Model segmentation and finite element analysis. The ultra-high resolution MIDA model was adapted for analysis in this study. **(A)** Skin tissue mask with periorbital electrodes (gray: electrode; blue: sponge). **(B)** The modeled brain, cranial nerves, blood vessels, eye structure, optic nerves, and electrodes (both active periorbital and the return inion electrode shown). **(C)** Zoomed view corresponding to the dashed section in panel **(B)** highlighting segmentation detail in the region of interest. Finite element analysis of current flow produced by rACS: Induced electric field magnitude plots on the cortical and eye level perspective **(D)** and bottom view **(E)**. A representative axial 2D cross-section view of electric field magnitude following the retinofugal tract was chosen and plotted **(F).** Panel **(G)** shows the induced electric field on the eyes and optic nerve. Panel **(H)** shows the rear view. Panel **(I)** shows the primary visual cortex (V1) corresponding to the dashed section in panel **(H)**. A representative 2D axial cross-section view of electric field magnitude taken at the level of half of the visual cortex along the superior–inferior plane is shown in panel **(J)**.

The stimulation electrodes were created as CAD files mimicking the exact physical geometry and dimensions of the electrodes used in the experiments. The electrodes were positioned interactively within the image data simulating the electrode montage used for rACS (see Figure 1C). The adaptive meshes derived from the segmentation masks were then imported into COMSOL Multiphysics (Burlington, MA, United States) for finite element computation. The final model comprised >10 million elements with >15 million degrees of freedom.

The representative isotropic average electrical conductivities assigned to the different tissue compartments and the electrode materials (in S/m) are listed in Table 1.

**TABLE 1:** Assigned electrical conductivities.

**Tissue compartment/Electrode material**	**Electrical conductivity (S/m)**
Scalp	0.465
Muscle	0.35
Skull	0.01
CSF	1.65
Gray Matter	0.276
White Matter	0.126
Fat	0.04
Blood vessels	0.7
Eye Lens	0.32
Eye Retina/Choroid/Sclera	0.623
Eye Vitreous	1.55
Eye Cornea	0.5
Eye Aqueous	1.5
Optic Tract/Optic Chiasm/Cranial Nerve II	0.126
Air	1.00E−07
Sponge (felt buffer)	1.84
Periorbital electrode (gold electrodes)	4.10E+07
Inion electrode (stainless steel)	1.45E+06

*The representative isotropic average electrical conductivities assigned to the different tissue compartments and the electrode materials (in S/m).*

The Laplace equation was solved and current densities corresponding to 350 μA total current were applied at the anode or active electrode (s). Ground was applied at the return electrode and all other external surfaces were treated as insulated. The linear iterative solver of conjugate gradients with a relative tolerance of 1E-6 was used.

Surface as well as cross-sectional EF magnitude maps on the gray matter, retina, and the optic nerve were obtained. For the scalp, the surface current density magnitude plot was obtained.

### Questionnaire

The questionnaire we employed is based on the one proposed by Brunoni et al. (2011) and investigated the presence of headaches, difficulties in concentrating, acute mood changes, visual perceptual changes, fatigue and discomforting sensations tingling, itching and/or burning under the electrodes during and after rACS, as well as PS. The item “Difficulties in concentrating” was defined in accordance with Montgomery and Asberg (1979), while the item “Fatigue” was defined in accordance with Chaudhuri and Behan (2004).

We modified the questionnaire by adding a description of phosphenes. Furthermore, to assess the overall tolerability, we defined the broad category of “Pain” as a summary of all discomforting sensations mentioned above and added a Numeric Rating Scale (NRS-11, 11 stages from 0 to 10, 10 being the strongest imaginable pain and 0 the absence of pain) (Farrar et al., 2001) as a more in-depth and reliable measurement (Downie et al., 1978). We discarded the four-point intensity rating for the other categories to avoid a “halo effect” bias (Streiner and Norman, 2008). We assumed that the foreign body feeling reported for physiologically similar transcorneal electrical stimulation (TCES) came from the electrode lying directly on the cornea (Gekeler et al., 2006) and therefore decided not to include it.

Three months after stimulation, the subjects received a second questionnaire to identify late and longer lasting after-effects.

As the data is not normally distributed and equal variance of residuals cannot be assumed, the severity of pain was analyzed in Wilcoxon Signed Ranks Tests for paired samples. The nominally scaled side effects were analyzed in Fishers Exact Tests, as expected values in several of the cells of a contingency table are below the recommended threshold for a classical Chi-Squared Test (Larntz, 1978). *P*-values of <0.05 were considered significant. All analyses were performed using IBM SPSS Statistics, Version 19.0.0.1 (IBM, United States).

## Results

### Stimulation Parameters

An average 10 Hz phosphene threshold at 290.50 μV (SD 45.36), impedances at 12.05 kΩ (SD 2.89), and an average amplitude of 351.69 μA (SD 63.95) were noted. Calculated from peak-to-peak amplitude, the current density at electrode level amounted to a mean 1.00 mA/cm^2^ (SD 0.28), and the charge density to 0.60 C/cm^2^ (SD 0.11). As sine waves pulses were employed, we additionally calculated the effective amplitude, resulting in a mean 248.68 μA (SD 47.0). Using effective amplitude, current density amounted to a mean 0.71 mA/cm^2^ (SD 0.13), and the charge density to 0.42 C/cm^2^ (SD 0.08). RACS was found to be well within safety limits and the findings comparable to other similar stimulation methods (see Table 2). Regarding stimulation amplitude, rACS (0.35 mA) was comparable to most TCES and tES montages (ranging from 0.08 to 1.2 mA). Electrosleep and the maximum intensity stimulation employed by Gekeler et al. (2006) were found to employ higher amplitudes (3–25 mA). The stimulated area (0.35 cm^2^) is smaller than most tES montages (16–35 cm^2^), comparable only to Electrosleep and TCES (0.35–1.25 cm^2^). Regarding stimulation frequencies, rACS was compared to studies using similar frequencies (10–20 Hz), with the exceptions of Electrosleep, which is set at higher frequencies (100 Hz) as well as the non-oscillating tDCS and Gekeler’s TCES. The calculations following these observations place the charge density of rACS just above the TCES of Ma et al. (2014) and far below the safety limit published by Liebetanz et al. (2009). This is consistent for charge per phase and charge density per phase. Regarding current density, rACS (1 mA/cm^2^) ranks below Ma (1.2 mA/cm^2^), well below the maximum intensity employed by Gekeler (8.57 mA/cm^2^) and far below the safety limit proposed by McCreery (25 mA/cm^2^). These findings are even more pronounced when using effective amplitude.

**TABLE 2:** Comparison of stimulation parameters.

	**Amplitude**	**Area**	**Duration**	**Frequency**	**Current density**	**Charge density**	**Charge per phase**	**CD per phase**
	**(mA)**	**(cm^2^)**	**(min)**	**(Hz)**	**(mA/cm^2^)**	**(C/cm^2^)**	**(C/ph)**	**(C/(cm^2∗^ph))**
Safety limits (Agnew and McCreery, 1987)	–	–	–	–	25	–	–	0.000400
Safety limits (Liebetanz et al., 2009)	0.5	0.035	10	–	14.29	85.714	–	–
						(52.400)		
rACS	0.35	0.35	10	10	1	0.599	0.000035	0.0001
rACS (effective amplitude)	0.25	0.35	10	10	0.71	0.423	0.000025	0.000071
Electrosleep (Sergeev, 1963)	25	1.25	60	100	20	72	0.00025	0.0002
TCES (Ma et al., 2014)	1.2	1	5	20	1.2	0.36	0.00006	0.00006
TCES (Delbeke et al., 2001)	0.28	1.25	7	10	0.22	0.094	0.000028	0.000022
TCES (Gekeler et al., 2006) max	3	0.35	7	–	8.57	–	–	–
TCES (Gekeler et al., 2006) optimal	0.08	0.35	7	–	0.22	–	–	–
tACS (Antal et al., 2008)	0.4	16	5	10	0.03	0.008	0.00004	0.000003
tSDCS (Paulus et al., 2013)	0.25	16	4	10	0.02	0.004	0.000025	0.000002
tDCS (Nitsche et al., 2003)	1	35	9	–	0.03	–	–	–

*Current density measures at electrode level employed for stimulation types employing continuous current (TCES (Gekeler) and tDCS), charge density, charge per phase and charge density (CD) per phase for oscillatory stimulation (rACS, Electrosleep, TCES (Ma and Delbeke), tACS and tSDCS).*

### Finite Element Modeling

The EF distributed by rACS is strongest at the eye level, with the highest current density estimates at the retina. Further areas of elevated current densities are optic nerve and cortex (Figures 1A,B).

The calculated maximum current density at the retina amounted to a maximum of 1.24 A/m^2^, while optic nerve (0.33 A/m^2^) and cortex (0.13 A/m^2^) were both subjected to less current flow. Regarding the EF, we estimated a maximum of 2.6 V/m in the optic nerve, followed by 1.99 V/m for the retina and 0.47 V/m for the cortex. Finally, current density at skin level underneath the active electrode amounted to a maximum induced value of 14.79 A/m^2^ (Figure 1C), with the EF estimated at 31.80 V/m. It should be noted that due to edge effects, the observed values are higher than the current density toward the middle of the electrode which is simply the current injected over the contact area. For a detailed view, see Table 3.

**TABLE 3:** Modeling data and comparison to safety limits.

		**Current density**	**Electric field**
		**(mA/cm^2^)**	**(V/m)**
Safety limits	Liebetanz et al., 2009	14.29	42
rACS (retina)	Max	0.124	1.99
	Mean	0.007	0.11
	Median	0.005	0.08
rACS (optic nerve)	Max	0.033	2.6
	Mean	0.003	0.2
	Median	0.002	0.14
rACS (cortex)	Max	0.013	0.47
	Mean	0.001	0.05
	Median	0.001	0.04
rACS (V1)	Max	0.003	0.12
	Mean	0.001	0.03
	Median	0.001	0.03

*This table presents the finite element modeling results for rACS: Current density and electric field estimates for retina, optic nerve, cortex and specifically the primary visual cortex (V1) in comparison to reported safety limits.*

### Adverse Events

Table 4 summarizes the adverse events in the 20 rACS and PS sessions in healthy participants. None of the subjects requested the stimulation to be terminated or required medical attention. In their subjective reports, rACS associated adverse events were predominant during stimulation, and PS associated adverse events were predominant following stimulation. More explicitly, a tingling sensation occurred in 70% of the subjects during but not after rACS. An itching sensation under the electrodes was reported by 30% of the subjects during rACS and 25% after rACS. A burning sensation was felt by 30% of the participants during but not after rACS. Fatigue occurred during, as well as after, stimulation in 35 and 20% of the rACS and PS group, respectively. Headaches were reported only by PS participants during stimulation (15%). After stimulation, it was reported by 5% for both PS, as well as rACS participants. Difficulties in concentrating were reported by 10% of the participants after PS, but not after rACS. There were no cases of acute mood changes, nausea and visual perceptual changes or lasting adverse events 3 months after stimulation.

**TABLE 4:** Adverse events for rACS and PS.

		**rtACS**		**Photic Stim**	
		***n***	**%**	***n***	**%**
Pain (overall)	During	8	40	4	20
	After	0	0	2	10
Fatigue	During	7	35	4	20
	After	7	35	4	20
Tingling	During	14	70	0	0
	After	0	0	0	0
Headache	During	0	0	3	15
	After	1	5	1	5
Itching	During	6	30	0	0
	After	5	25	0	0
Burning	During	6	30	0	0
	After	0	0	0	0
Difficulties in Concentrating	During	0	0	0	0
	After	0	0	2	10
Metallic Taste	During	3	15	0	0
	After	0	0	0	0
Muscle twitches	During	3	15	0	0
	After	0	0	0	0
Acute mood changes	During	0	0	0	0
	After	0	0	0	0
Nausea	During	0	0	0	0
	After	0	0	0	0

*Reports of adverse events during and after stimulation in subjects and percent (data rounded to integers).*

### Pain

**FIGURE 2:**
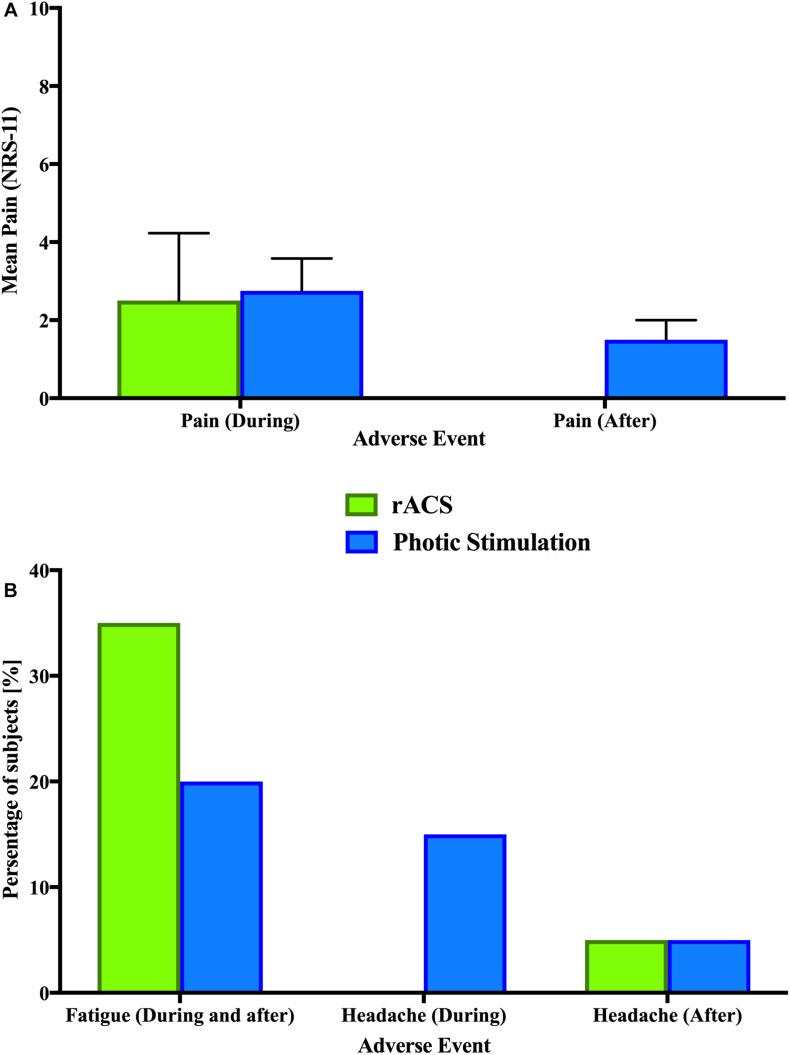
Adverse events. A comparison of adverse events between rACS (green) and PS (blue). None of the depicted differences were significant in Bonferroni-corrected multiple comparisons. **(A)** Depicted is the mean rating (NRS-11) of overall pain and discomfort in affected subjects during and after stimulation. Error bars represent the standard deviation. **(B)** Comparison of shared adverse events (fatigue and headache) in percentage of subjects.

Forty-percent of the subjects reported pain (Figure 2A) during rACS (mean intensity 2.5, SD 1.73) and 20% during PS (2.75, SD 0.83). While none of the participants reported pain after rACS, this was the case for 10% after PS (1.5, SD 0.5).

### rACS vs. Photic Stimulation

In the statistical analyses, rACS and PS showed no significant effect of stimulation type (rACS versus PS) on pain intensity as assessed by Wilcoxon Signed Ranks Tests (Figure 2A), fatigue, headache and difficulties in concentrating as assessed by Fisher’s Exact Tests (Figure 2B) during as well as after stimulation. PS and rACS significantly differed regarding skin sensations of tingling, itching and burning (*P* < 0.05, Fisher’s Exact Tests), which all occurred exclusively in rACS. For a more detailed view, see Tables 5, 6. The full dataset behind this comparison is available as Supplementary Material.

**TABLE 5:** Results of the Wilcoxon-signed ranks tests.

**Source**			***Z***	**Asymp. Sig. (2-tailed)**
Stimtype (rACS vs. PS)	Pain (overall)	During	–0.987^a^	0.323
		After	–1.342^b^	0.180

*The results of the statistical analysis on overall pain during and after stimulation. ^a^Based on positive ranks. ^b^Based on negative ranks.*

**TABLE 6:** Results of the Fisher’s exact tests.

**Source**			**Exact Sig. (2-tailed)**
Stimtype (rACS vs. PS)	Fatigue	During	0.480
		After	0.480
	Difficulties in Concentrating	During	–
		After	0.487
	Headache	During	0.231
		After	1.000
	Itching	During	0.020^∗^
		After	0.047^∗^
	Burning	During	0.020^∗^
		After	–
	Tingling	During	0.000^∗^
		After	–

*The results of the statistical analysis on the nominally scaled adverse events presented in Table 4. The asterisk (^∗^) marks significant results.*

## Discussion

To address the safety profile of rACS, we assessed theoretical safety limits as well as finite-element modeling data and compared the reported adverse events for rACS and simple PS.

The primary findings are that rACS is safe based on the following observations: (1) stimulation parameters (current and charge densities at the electrode) are within theoretical safety limits, (2) finite element modeling data shows the same for EF estimates and current densities at eye, retina and cortex, and (3) adverse events are comparable to PS in direct experimental comparison (see Tables 3, 4) and rate as well as severity of adverse events did not exceed that of other established brain stimulation methods (see Table 2).

### Stimulation Parameters

To be efficacious and safe, a stimulation system must stimulate neural tissue without damaging tissue or electrode. Tissue damage is caused when excitable tissue is overstimulated and electrode damage ensues as metal oxidation occurs (Peterchev et al., 2012). Current density and charge density have been proposed as predictors for such damage (Bikson et al., 2016).

#### Current Density

Current density is the proposed optimal safety parameter for a constant current stimulation (Nitsche et al., 2003) and can be derived from the effective amplitude and compared to safety limits (Agnew and McCreery, 1987) as well as other similar stimulation paradigms (Gekeler et al., 2006; Ma et al., 2014).

We find that rACS current densities are within reported safety limits for tissue damage (Yuen et al., 1981; Lindenblatt and Silny, 2002; Liebetanz et al., 2009; Gellner et al., 2016).

#### Charge Density and Charge per Phase

While current density is a well-established safety parameter, it is best suited for assessing the safety of constant current stimulation. ACS injects less charge than constant current stimulation of the same amplitude (Liebetanz et al., 2009; Schmidt et al., 2013a), dependent on stimulation frequency and duty cycle (Chaieb et al., 2014). The safety limits of charge balanced ACS, such as rACS, are therefore more precisely determined by charge density and charge per phase (Nitsche et al., 2003; Merrill et al., 2005).

We find that rACS charge densities are also within reported safety limits for tissue damage (Yuen et al., 1981; Lindenblatt and Silny, 2002; Liebetanz et al., 2009; Gellner et al., 2016; Jackson et al., 2017).

#### Comparison to Other Stimulation Types

While stimulating at higher current and charge densities than most forms of tES, rACS stimulation parameters proved comparable to dose parameters reported for TCES using up to 10 mA per pulse to establish safety guidelines (Gekeler et al., 2011), well below early montage parameters for both stronger and longer stimulation used in early studies addressing Electrosleep therapy (Robinovitch, 1914; Knutson, 1967; Peterchev et al., 2012), and well below current densities reported for stimulation via implanted self-sizing spiral cuff electrodes in blind patients over the course of several years (Delbeke, 2011) (see Table 2).

Despite arguable differences between different stimulation techniques, there are remarkable similarities, e.g., comparably distant periorbital montage of electrodes, as well as modeling results for the serial resistance of the skin and eyelid (Delbeke et al., 2000; Merrill et al., 2005; Gekeler et al., 2006) to motivate this comparison.

This leads to the conclusion that the employed charge injection was safe with regards to possible tissue as well as electrode damage. In the future, studies addressing the calculation of rheobase and chronaxie and stimulation with variable pulse parameters might help to further reduce charge injection to the minimum necessary to efficaciously achieve a neuronal response (Irnich, 1980, 2010; Delbeke et al., 2001).

### Finite Element Modeling

#### Electric Field Distribution

Expectedly, the EF distribution shows a clear focus on retina and optic nerve, while the cortical electric current flow is much weaker. Due to the electrode montage being superior***–***inferior, we see stronger EFs in the temporal regions and at the return electrode. While there is increased flow through the subcortical structures, brain stem and cerebellum, there appears to be no strong current flow to occipital areas, with a maximum current density of 0.033 A/m^2^ and a maximum EF of 0.1208 V/m (Table 3 and Figure 1).

This confirms the retinofugal pathway as the primary target of rACS. Still, stimulation intensity should be closely monitored, as strong over-threshold stimulation might lead to unwanted effects on subcortical structures.

#### Current Densities and EF Estimates

Evidence from relevant animal models indicates that brain injury by tDCS occurs at predicted brain current densities (14.9 A/m^2^) (Liebetanz et al., 2009; Gellner et al., 2016; Jackson et al., 2017). Considering the well-established threshold proposed by Liebetanz et al. (2009), rACS maximum current densities rank two orders of magnitude (OOM) below lesion threshold for retina and optic nerve and three OOM below for the cortex.

Additionally, all of the EF estimates are at least one OOM below the safety threshold of 42 V/m (Liebetanz et al., 2009; Gellner et al., 2016; Jackson et al., 2017). It should be noted that, as mentioned above, ACS injects less charge than constant current stimulation of the same amplitude (Liebetanz et al., 2009; Schmidt et al., 2013a), and we calculated the current densities from peak-to-peak amplitude instead of effective amplitude. The risk of damage will consequently rather be over-than underestimated. We therefore conclude the rACS employed in this study should be safe from a modeling standpoint as well.

### Adverse Events

No fatal or serious adverse events (Wester et al., 2008) were observed for rACS. The most notable adverse events in the present study were tingling, burning, itching and fatigue. The hazard rate for these adverse events is to be considered “very common” (>1/10 cases). This is comparable to results from other forms of tES (Brunoni et al., 2011), suggesting for tDCS that the type of adverse event is mild and their frequency of occurrence is “common.” Direct experimental evidence shows significantly more cutaneous adverse events, but significantly less concentration deficits after stimulation for rACS as compared to PS (Table 5).

As the modeling results showed high maximum current densities and EF estimates at skin level, the presence of cutaneous adverse events during and after rACS comes as no surprise. Comparing rACS and PS regarding the summary category of pain, we have to note the complete lack of cutaneous sensations in PS and that multiple aversive sensations may be clustered and perceived in sum total as painful (Tuckett, 1982).

#### Skin Rashes and Damage

None of the subjects reported skin rashes or damage. Whereas the applied charge density is clearly strong enough to stimulate C-nociceptors, it is too low and the duration is too short to induce skin damage (Dzhokic et al., 2008). For direct current stimulation, it has been shown that 1 mA via two 7 × 5 cm rubber electrodes in over 2000 stimulation sessions (Loo et al., 2011) can be applied for 20 min with no skin damage. Again, ACS is less likely than direct current stimulation to induce tissue or electrode damage. Although rACS is unlikely to induce skin damage, this study adhered to previous suggestions for avoiding cutaneous adverse events (Loo et al., 2011).

#### Tingling, Itching and Burning

Electrical stimulation of skin nociceptors is known to produce itching, burning and tingling sensations in the animal model, as well as in human subjects (Jarvis and Voita, 1971; Tuckett, 1982; Kellogg et al., 1989; Ledger, 1992). While even persisting shortly after stimulation due to central processes, these sensations are not necessarily indicative of local damage induced by stimulation (Tuckett, 1982).

#### Pain

One third of the subjects reported pain with a median strength of 2.5 NRS. The sensation of pain during and after electric stimulation is understood to be a combination of several factors, with the terminal branches of C-nociceptors of the stimulated skin acting as the primary central conductor (Magerl et al., 1987; Garnsworthy et al., 1988; Hakkinen et al., 1995). This matches subject descriptions of deep and spread pain associated with itch and burning sensations in this study (six cases) as well as anecdotal reports of painful perceptions that could not be attenuated by topical anesthetic and the lack of radiating pain sensations reported elsewhere (Hakkinen et al., 1995). Due to the common occurrence of cutaneous sensations, topical anesthesia might be preferential especially for placebo control or rACS versus PS studies. This study did not use topical anesthesia, as it might mask development of skin damage.

While the feeling of pain and discomfort should be monitored closely in future studies, it should be noted that we found no significant difference between rACS and well-established and tolerable PS regarding overall discomfort/pain (Table 5).

This pain during and after PS is most likely a form of “discomfort glare” associated with visual discomfort, annoyance, irritability or distraction without affecting the ability to see, but leading to symptoms of visual fatigue (Ticleanu and Littlefair, 2015).

#### Phosphenes

As we stimulated our subjects at 120% phosphene threshold, all subjects experienced phosphenes. These phosphenes induced by rACS were typically described as flickering at the edges of the field of view and not experienced as painful.

Historically, phosphenes induced by alternating current have been seen as a purely retinal phenomenon (Rohracher, 1935) resulting from the high susceptibility of the retina to electricity (Ziemssen, 1864). For rACS and other forms of tES the amount of confounding retinal or cortical stimulation following low-voltage stimulation is unknown or a matter of controversy (Paulus, 2010).

Yet, due to the respective montages there should be a magnitude of difference between methods (Peterchev et al., 2012) with TCES inducing the most, rACS with periorbital-occipital montages intermediate, and tES the least retinal stimulation (Delbeke et al., 2001; Thil et al., 2007; Paulus, 2010).

A previous tACS modeling effort indicated why transcranial stimulation may induce retinal phosphenes (Laakso and Hirata, 2013) by virtue of current density induced in the eyes exceeding phosphene thresholds. As different electrode montages result in different current flow patterns, whether a particular montage would result in retinal phosphenes would naturally depend on the montage being studied. Specifically they show that the threshold for retinal phosphenes for commonly used tACS montages is exceeded with stimulation current of 500–1000 μA (depending on the montage considered). Another prior tACS/tDCS modeling effort demonstrated that bilateral montages result in not only more focused current flow but higher current intensities than midline montages (Neuling et al., 2012). While no detailed analysis is performed on the eye regions, the authors state that the closer one of the stimulation electrodes is to the eye regions, the easier it is to perceive phosphenes.

Where exactly rACS phosphenes are generated remains subject to further investigation. While we find the highest EF estimates in the optic nerve, other authors (Brindley, 1955; Ma et al., 2014) suggested bipolar cells, or the parts of rod and cone cells lying inside the external limiting membrane as the main site of stimulation. In line with the flickering at the edges of the field of view as reported by our subjects for rACS at 120% phosphene threshold, it can be argued that inner retinal neurons are the most probable site at which an electrical stimulus exerts its primary effect, with predominant activation of the peripheral retina (Ma et al., 2014). This adds further support to previous findings suggesting that the primary location of the majority of retinal damage (the retinal pigment epithelium, RPE) induced by photochemical noxae is bypassed by electrical stimulation (Grützner et al., 1958). Besides fatigue and cutaneous effects, the participants described more phosphene or light related adverse events in association with well-known and safe PS applied at 120% light threshold than with rACS applied at 120% phosphene threshold.

#### Fatigue

Fatigue, reported by one third of the subjects after rACS, has been suggested in previous research to be an unspecific effect of tES. Similar to rACS, the early approaches to tES involved two “active” electrodes placed directly over the eyes, presumably to facilitate active current delivery through the optic foramina. These montages were first used in Electrosleep research initiated in Robinovitch (1914), with extensive research following (Obrosow, 1959; Sergeev, 1963; Brown, 1975). The consensus after about 60 years was that Electrosleep induces unspecific sleepiness and fatigue related to stimulation (Guleyupoglu et al., 2013).

The findings in this study, that rACS produces more fatigue than PS, support the notion of an indirect and unspecific central (adverse) effect specific to electrical stimulation. This notion is in line with previous findings showing that action potentials induced by electrical stimulation of the retina can propagate directly to the visual cortex (Grützner et al., 1958), produce different evoked potentials (Potts et al., 1968) and modulate central rhythms (Schmidt et al., 2013a) as well as large scale networks of the brain (Bola et al., 2014).

#### CNS Damage and Seizure Risk

Beyond fatigue, the possibility of direct structural damage to central nervous structures by rACS seems low considering the distance between charge injection and brain tissue as well as stimulation strength. Yet, for rhythmic PS the danger of inducing an epileptic seizure is well established. Although not found in this study, for electrical stimulation the danger must also be assumed to be high due to neurophysiological similarities with intermittent photic stimulation (IPS) (Brindley, 1955) and proven effects on central processes and neural synchrony (Parra et al., 2003). Additionally, although no reports of seizures after comparable electrical stimulation sessions exist (Brunoni et al., 2012), we will continue to employ photosensitivity and epilepsy as exclusion criteria for future rACS studies.

## Conclusion and Outlook

Having theoretically and experimentally characterized the relative safety profile of rACS, we believe future studies can further investigate retinal mechanisms of action for ACS effects, especially in comparison with tACS. Additionally, rACS allows for studies addressing the interaction of different signal types entering the visual system through two separate input channels (left and right eye) and converging at the level of the primary visual cortex. This provides an promising tool for studies aiming to address a common framework of action for NiBS with more than one input-signal, e.g., noise and oscillation (Schmidt et al., 2013b).

## Data Availability

The datasets generated for this study are available on request to the corresponding author.

## Ethics Statement

All protocols conformed to the Declaration of Helsinki, and were approved by the Ethics Committee of the Charité – Universitätsmedizin Berlin (“Ethikkommission der Charité – Universitätsmedizin Berlin”). Informed consent was obtained from all individual participants included in the study. This study adheres to the principles of good scientific practice of the Charité – Universitätsmedizin Berlin (“Grundsätze der Charité zur Sicherung guter wissenschaftlicher Praxis”).

## Author Contributions

LH, AD, SS, MS, and SB conceived and designed the study. LH, AD, CT, AJ, and SS carried out data acquisition and analysis. LH, AD, and SS drafted the manuscript. CT, AJ, AK, MR, MS, and SB critically revised the manuscript. All authors participated in the interpretation of the data.

## Conflict of Interest Statement

The authors declare that the research was conducted in the absence of any commercial or financial relationships that could be construed as a potential conflict of interest.

## Abbreviations

EF: electrical field
NiBS: non-invasive brain stimulation
NRS: numeric rating scale
PS: photic stimulation
rACS: retinofugal alternating current stimulation
RPE: retinal pigment epithelium
tACS: transcranial alternating current stimulation.
