# Supplementary material for: Safety Aspects, Tolerability and Modeling of Retinofugal Alternating Current Stimulation
Source: Front Neurosci. 2019 Aug 7;13:783. doi: 10.3389/fnins.2019.00783 (PMC6692662; doi:10.3389/fnins.2019.00783)
Supplement: Supplementary file 1 [file Data_Sheet_1.PDF]

Adverse Events Questionnaire
Nebenwirkungsfragebogen

Study/*Studie*:

Stimulation-ID/*Stimulations-ID*:

Subject-ID/*Probanden-ID*:

Date/*Datum*:

Did you experience any of the following symptoms or side effects during or after stimulation?
Haben Sie folgende Symptome oder Nebenwirkungen während oder nach der Stimulation bemerkt?

	**During/*Während***	**After/*Nach***	**No/*Nein***
Fatigue/*Erschöpfung*	☐	☐	☐
Headache/*Kopfschmerz*	☐	☐	☐
Difficulties in Concentrating/*Konzentrations-schwierigkeiten*	☐	☐	☐
Tingling/*Kribbeln*	☐	☐	☐
Itching/*Jucken*	☐	☐	☐
Burning/*Brennen*	☐	☐	☐
Acute Mood Changes/*Akute Stimmungsschwankungen*	☐	☐	☐
Nausea/*Übelkeit*	☐	☐	☐
Visual Perceptual Changes/ *Änderung der Wahrnehmung*	☐	☐	☐
Pain/*Schmerz*	☐	☐	☐

If you experienced pain, how strong would you rate it on a scale of 0-10, 0 being the absence of pain and 10 being the strongest pain imaginable?
Falls Sie Schmerzen verspürt haben, wie stark würden Sie diese auf einer Skala von 0-10 bewerten, mit 0 als Abwesenheit von Schmerz und 10 als stärkstem vorstellbaren Schmerz?

	**During/*Während***	**After/*Nach***
Pain Rating/*Schmerzstärke* (0-10)		

Thank you for your cooperation!
Danke für Ihre Mithilfe!
