# Supplementary material for: Safety Aspects, Tolerability and Modeling of Retinofugal Alternating Current Stimulation
Source: Front Neurosci. 2019 Aug 7;13:783. doi: 10.3389/fnins.2019.00783 (PMC6692662; doi:10.3389/fnins.2019.00783)
Supplement: Supplementary file 2 [file Data_Sheet_2.PDF]

Adverse Effects Questionnaire – Follow-up
Nebenwirkungsfragebogen - Verlaufskontrolle

Study/*Studie*:

Stimulation-ID/*Stimulations-ID*:

Subject-ID/*Probanden-ID*:

Date/*Datum*:

Which, if any, of the following symptoms or side effects outlasted the stimulation session and persisted until now?
Welches, soweit zutreffend, der folgenden Symptome oder Nebenwirkungen hat die Stimulationssitzung überdauert und bis jetzt angehalten?

Fatigue/*Erschöpfung*	☐
Headache/*Kopfschmerz*	☐
Difficulties in Concentrating/*Konzentrationsschwierigkeiten*	☐
Tingling/*Kribbeln*	☐
Itching/*Jucken*	☐
Burning/*Brennen*	☐
Acute Mood Changes/*Akute Stimmungsschwankungen*	☐
Nausea/*Übelkeit*	☐
Visual Perceptual Changes/ *Änderung der Wahrnehmung*	☐
Pain/*Schmerz*	☐

If you experienced pain, how strong would you rate it on a scale of 0-10, 0 being the absence of pain and 10 being the strongest pain imaginable?
Falls Sie Schmerzen verspürt haben, wie stark würden Sie diese auf einer Skala von 0-10 bewerten, mit 0 als Abwesenheit von Schmerz und 10 als stärkstem vorstellbaren Schmerz?

	**During/*Während***	**After/*Nach***
Pain Rating/*Schmerzstärke* (0-10)		

Thank you for your cooperation!
Danke für Ihre Mithilfe!
